# Investigation into the Enhancement Effects of Combined Bioremediation of Petroleum-Contaminated Soil Utilizing Immobilized Microbial Consortium and Sudan Grass

**DOI:** 10.3390/toxics13070599

**Published:** 2025-07-16

**Authors:** Tie-Jun Wang, Zi-Yue Ding, Zi-Wei Hua, Zi-Wang Yuan, Qiu-Hong Niu, Hao Zhang

**Affiliations:** 1 Henan Province Engineering Technology Research Center of Animal Disease Control and Prevention, Nanyang Vocational College of Agriculture, Nanyang 473000, China; tie20062002@163.com; 2 College of Life Science, Research Center of Henan Provincial Agricultural Biomass Resource Engineering and Technology, Nanyang Normal University, Nanyang 473061, China; 15328331055@163.com (Z.-Y.D.); 15239943441@163.com (Z.-W.H.); ziwang0329@outlook.com (Z.-W.Y.)

**Keywords:** petroleum contamination, petroleum degrading strains, immobilized microbial consortium, Sudan grass, combined bioremediation

## Abstract

Petroleum-contaminated soil is an increasingly severe environmental issue. The integration of phytoremediation and microbial remediation can effectively mitigate their individual limitations and enhance remediation efficiency. In this study, four newly isolated bacterial strains (including *Cytobacillus* and *Rhodococcus*) that exhibited preferential degradation of distinct petroleum components were combined with the rhamnolipid-producing strain *Pseudomonas aeruginosa* SL-1. The immobilization of this petroleum-degrading microbial consortium was performed by biochar adsorption and sodium alginate embedding, subsequently optimized using response surface methodology (0.75 g·L^−1^ of biochar, 40 g·L^−1^ of sodium alginate, and 40 g·L^−1^ of calcium chloride). The results showed that the highest petroleum degradation rate (97.1%) of immobilized bacterial consortium was achieved at 72 h at a petroleum concentration of 5.0 g·L^−1^. When combined with Sudan grass for soil bioremediation, the degradation rate reached 72.8% after 120 d for soil containing 5.0 g·kg^−1^ of petroleum, higher than the results for the treatments with only immobilized bacterial consortium (53.0%) or Sudan grass (49.2%). Furthermore, significant improvements were observed for soil pH; nitrogen, phosphorus, and potassium contents; and urease, dehydrogenase, and catalase activities. Composite treatment also significantly increased the diversity and richness of the soil bacterial community and regulated its structure, function, and network composition. This study offers theoretical insights and potential practical applications for the enhanced bioremediation of petroleum-contaminated soils.

## 1. Introduction

Petroleum is the most dominant energy source used to meet the burgeoning energy demands of humanity [1]. Petroleum plays an indispensable role in meeting the ever-growing global needs for powering industries, transportation, and daily life. The large-scale extraction from petroleum fields and the extensive transportation networks across the globe have transformed petroleum pollution into a pressing global environmental concern [2]. Over its entire lifecycle, which encompasses extraction, refining, storage, transportation, and utilization, a substantial quantity of petroleum finds its way into soil [3]. This influx far exceeds the natural self-purification capacity of the soil, leading to a breakdown in its normal functions, such as nutrient cycling and water infiltration. This not only poses a threat to soil ecosystems, but also has far-reaching implications for water resources and human health. Studies have shown that the petroleum content of soil surrounding petroleum wells often exceeds 500 mg·kg^−1^, with a contamination range of 1000–2000 m^2^. Furthermore, approximately 80% of petroleum pollutants are retained within the top 50 cm of the soil, thereby severely affecting plant growth and threatening ecological safety [4,5,6]. Additionally, petroleum contamination disrupts the soil structure, decreases permeability, and creates localized high-concentration pollution, ultimately disturbing the balance of carbon, nitrogen, and phosphorus and weakening soil functionality [7,8,9]. Thus, petroleum-contaminated soil has become a significant global environmental issue, with increasing academic attention focused on remediation strategies [10].

Bioremediation, with its low cost and lack of secondary pollution [11], has become a primary remediation method. Various strategies include microbial bioremediation, phytoremediation, and combined plant–microorganism remediation [12,13]. However, single-microorganism bioremediation is limited by soil conditions, and the survival time of exogenous microorganisms is restricted. Single-plant remediation requires long recovery periods, limiting its application [14]. Combined plant–microorganism remediation takes advantage of the cost-effectiveness and operational ease of phytoremediation, while benefiting from the shorter cycles and complete petroleum-degrading metabolic capabilities afforded by microorganisms [15,16]. Using *Stutzerimonas stutzeri* and *Pseudomonas sundara* with marigold plants, Khilji et al. demonstrated the successful combined remediation of soil contaminated with 105.5 mg·kg^−1^ of cadmium, 170.2 mg·kg^−1^ of lead, and 75.32 mg·kg^−1^ of chromium, reducing the metal contents to 22.63, 23.39, and 11.47 mg·kg^−1^, respectively, after 60 d [17]. However, the hydrophobicity and low solubility of petroleum hydrocarbons make them prone to adsorption by organic matter in the soil, decreasing their availability to microorganisms [18,19]. As a biosurfactant, rhamnolipids promote the migration of pollutants from the solid to liquid phase, forming stable emulsions that enhance pollutant bioavailability and significantly improve degradation efficiency [20,21]. Mottola et al. demonstrated that nanoemulsions of synthetic rhamnolipids effectively induced plant immune responses without damaging plant tissues or negatively affecting native soil microbiota and beneficial soil-borne microbes, supporting their ecological safety for agricultural applications [22]. Guo et al. demonstrated that rhamnolipids enhanced the bioavailability of cadmium by altering the soil physicochemical properties and microbial community, thereby promoting the remediation of cadmium-contaminated soil by *Sedum alfredii* [23]. Wang et al. showed that rhamnolipids effectively increased the bioavailability of polycyclic aromatic hydrocarbons (PAHs) by elevating the dissolved organic carbon concentration, and that when used in combination with mushroom substrate, the soil PAH degradation rate increased from 25.53% to 30.36% [24]. Furthermore, when free microorganisms are added to contaminated soil, their metabolism is influenced by the surrounding environment, limiting their survival and reducing their effectiveness in petroleum hydrocarbon biodegradation. Immobilized microorganism technologies, which fix free microorganisms onto carriers, reduce the environmental impact on their activity and enhance their pollutant degradation capacity [25,26]. Du et al. used sodium alginate (SA) and polyvinyl alcohol to encapsulate highly efficient pollutant-degrading strains, subsequently achieving over 80% chlorobenzene degradation within 72 h [27]. When studying heavy-metal-contaminated soil remediation, Yang et al. demonstrated that, compared to untreated groups, a biochar-immobilized microbial consortium improved root phenotypic traits by up to 2.4 times and significantly optimized the microbial community structure [28]. Dong et al. showed that a preparation of PEG-nZVI/GAC@B (polyethylene glycol-modified nano-zero-valent iron-loaded granular activated carbon-immobilized microorganisms) achieved a 96.30% 2,4-dichlorophenol removal rate in soil within 119 d. The combined adsorption-embedding immobilization technique effectively integrates the advantages inherent in both adsorption and embedding methods. Specifically, adsorption offers the benefit of the rapid attachment of microorganisms onto the carrier surface, with embedding providing a physical barrier that protects the microorganisms. Thus, by combining these two approaches, this technique significantly enhances the survival ability of free microorganisms. This, in turn, enables the microorganisms to better withstand harsh environmental conditions. Given its effectiveness in improving microbial performance, the combined adsorption-embedding immobilization technique shows great application potential in a range of fields, such as wastewater treatment, bioremediation, and industrial fermentation processes [29].

This study focused on addressing the challenges involved in petroleum remediation and aimed to establish an enhanced remediation system for petroleum-contaminated soils. A consortium of four efficient petroleum-degrading bacterial isolates and one rhamnolipid-producing strain was used as a basis for this work. The optimal degradation conditions were first determined by adjusting the bacterial consortium system, and then the immobilization process was optimized through response surface methodology. Next, suitable plants for use in petroleum hydrocarbon remediation were screened. The strengthening effect on petroleum-contaminated soil was evaluated by testing the remediation efficiency of the plant-only, immobilized consortium-only, and plant-immobilized consortium treatments, providing a theoretical and practical basis for the remediation of petroleum-contaminated soil.

## 2. Materials and Methods

### 2.1. Materials

LB medium, minimal salt medium (MSM), and sodium alginate (SA) solution were prepared following established protocols [30,31,32]. The rhamnolipid-producing strain *Pseudomonas aeruginosa* SL-1 was maintained in our laboratory. Other reagents and consumables were purchased from Nanjing Rexton Biotechnology Co., Ltd., Nanjing, China.

Polluted soil was collected from a petroleum field in the Central Plains region (35°42′ N, 114°59′ E, 5–20 cm) of China. After collection, the soil, categorized as sandy loam, was sieved through a 2 mm mesh screen and air-dried for later use.

### 2.2. Petroleum-Degrading Bacterial Isolation and Identification

To isolate petroleum-degrading bacteria, 10 g of long-term petroleum-contaminated soil was added to 100 mL of MSM, with petroleum as the sole carbon source. Enrichment was carried out at 30 °C, 200 r·min^−1^, for 7 d, and then 10% of the fermentation solution was transferred to fresh MSM. After three transfers, 200 μL of the enriched culture was spread onto solid MSM plates containing 5.0 g·L^−1^ petroleum to screen for different strains. The resulting colonies were inoculated into liquid LB medium and cultured to the logarithmic phase. Selected strains were transferred into liquid MSM with 5.0 g·L^−1^ petroleum, and incubated at 30 °C, 200 r·min^−1^, for 7 d. A control treatment was set up without bacterial inoculation. Petroleum extraction, qualitative gas chromatography analysis, and quantitative UV spectrophotometry were carried out according to the methods of previous studies [33,34,35]. Four efficient petroleum-degrading bacterial strains were isolated and named SY-2, SY-3, SY-4, and SY-5. Identification was based on the morphological, physiological, and biochemical characteristics, as well as phylogenetic analysis, as referenced in a previous study [36]. The physiological and biochemical characteristics of each strain were determined using the Biolog GEN III MicroPlate (Biolog, inc, Hayward, CA, USA), the Biolog PM3B Nitrogen Plate (Biolog, inc, Hayward, CA, USA), and the API ZYM kit (bioMérieux, S.A., Marcy-l’Étoile, France).

### 2.3. Petroleum-Degrading Microbial Consortium and Degradation Characteristics

Strains SY-2, SY-3, SY-4, SY-5, and SL-1 were inoculated in liquid LB medium at 30 °C and grown to the logarithmic phase. Six treatments were performed: single suspensions of 10 mL SY-2, SY-3, SY-4, and SY-5; microbial consortiums of each of the four strains with 2.0 mL of each inoculum and 2.0 mL sterile water (FH); microbial consortiums of each of the four strains with 2.0 mL of each inoculum and 2.0 mL of SL-1 suspension (FHSL-1). These inoculations were added to 100 mL of liquid MSM containing 5.0 g·L^−1^ of petroleum and incubated at 30 °C, 200 r·min^−1^, for 96 h. The petroleum concentration was quantified by UV spectrophotometry, and the degradation characteristics of the microbial consortiums with the highest degradation were further studied. The maximum absorption wavelength of the petroleum was determined by scanning a standard oil solution (1 mg·mL^−1^ in petroleum ether) from 220 to 300 nm using a UV spectrophotometer (Persee Analytics, Beijing, China), and the maximum was found at 238 nm. Standard solutions (3, 9, 15, 21, and 27 mg·kg^−1^) were used to generate a calibration curve by measuring the absorbance at 238 nm. Residual petroleum concentrations in the samples were determined using this curve. The petroleum degradation rate was calculated as follows: degradation rate (%) = (initial concentration − residual concentration)/initial concentration × 100%.

To determine the optimal temperature, 10 mL of bacterial suspension was added to 100 mL of MSM with 5.0 g·L^−1^ of petroleum and then incubated at 20, 25, 30, 35, and 37 °C, 200 r·min^−1^, for 96 h. Samples were collected every 24 h to measure the petroleum concentration. The optimal temperature was then determined. Similar evaluations were conducted to determine the effects of different petroleum concentrations (2.0, 3.0, 5.0, 7.0, and 9.0 g·L^−1^); pH (5.0, 6.0, 7.0, 8.0, 9.0, and 10.0); inoculum concentration (0.5, 1.0, 2.0, 3.0, 4.0, and 5.0%); and NaCl concentration (1.0, 2.0, 3.0, 5.0, and 7.0%). These experiments were conducted at 30 °C, 200 r·min^−1^, for 96 h, with the petroleum concentration measured every 24 h.

### 2.4. Preparation and Optimization of Immobilized Microbial Microspheres

Immobilized microbial microspheres were prepared by mixing 20 mL of mixed bacterial suspension (FHSL-1) with 1.0 g of biochar. The mixture was added to 100 mL of liquid MSM and incubated at 30 °C, 200 r·min^−1^, for 24 h. To evaluate the immobilization effect, the mixture was centrifuged, washed with phosphate buffer (Solarbio Life Sciences, Beijing, China), fixed with glutaraldehyde (Sinopharm Chemical Reagent Co., Ltd., Shanghai, China), dehydrated with ethanol (Sinopharm Chemical Reagent Co., Ltd., Shanghai, China), and subjected to critical point drying. The biochar structure was observed via scanning electron microscopy (SEM) (Hitachi SU8010, Hitachi High-Technologies, Tokyo, Japan) [37]. SA–biochar microspheres (immobilized microspheres) were formed by mixing 80 mL of SA solution (40 g·L^−1^) with 20 mL biochar microbial agent and then dropping the mixture into a 40 g·L^−1^ calcium chloride (CaCl_2_) solution for 24 h of cross-linking. After filtering, washing, and soaking in saline, the microspheres were stored at 4 °C.

Preparation of immobilized microspheres was then optimized using a single-factor experiment to study the effects of biochar (0.5, 0.75, 1.0, 1.25, and 1.5 g·L^−1^), SA (20, 30, 40, 50, and 60 g·L^−1^) and CaCl_2_ (20, 30, 40, 50, and 60 g·L^−1^) concentrations on the degradation efficiency of the formed microspheres. After 24 h of cross-linking, the microspheres were washed before further testing. Each group of microspheres was then incubated in 100 mL of MSM containing 5.0 g·L^−1^ of petroleum at 30 °C, 200 r·min^−1^, for 72 h, and the petroleum concentration was measured to calculate the degradation rate. Based on the results of this single-factor experiment, response surface methodology was used to optimize the preparation process.

### 2.5. Screening of Plants for Petroleum Remediation

Soil was collected (5–20 cm depth) from an experimental field at the Nanyang Normal University, China, and sieved through a 2 mm mesh screen. The soil was divided into 800 g portions. Petroleum was dissolved in n-hexane (Aladdin Reagent Co., Ltd., Shanghai, China) and mixed with the soil at four different concentrations: 0, 3.0, 5.0, and 7.0 g·kg^−1^. After standing for 2 d before receiving water, the soil was incubated for 7 d. The treated contaminated soil was then placed in pots and seeded with alfalfa, Sudan grass, and ryegrass, with 20 seeds per pot planted at a depth of 1.5 cm. The soil moisture content was maintained at 40%, with three replicates for each treatment. The pots were placed in a temperature-controlled growth chamber at 25 °C and under a 12 h light cycle. Germination was recorded daily, and after germination, the seedling height, root length, fresh weight, and dry weight were measured, along with the physical and chemical properties of the soil.

### 2.6. Combined Immobilized Microbial Consortium and Plant Remediation of Petroleum-Contaminated Soil

The combined effect of the immobilized microbial consortium and plants (Sudan grass) on the remediation of petroleum-contaminated soil was studied using a pot experiment. Each pot contained 2.0 kg of petroleum-contaminated (5.0 g·kg^−1^) soil. Five treatments were performed: CK (without petroleum-contaminated soil), OP (petroleum-contaminated soil), IM (immobilized microbial consortium), PR (plant remediation) and CP (combined microbial consortium + plant remediation). For the IM and CP treatments, the immobilized microbial consortium was mixed evenly with contaminated soil. For the PR and CP treatment, 15–30 Sudan grass seeds were planted at a depth of 1.5 cm, with 10 plants remaining per pot after thinning. The treatments were watered every 3 d to maintain 40% soil moisture. The petroleum concentration was measured on days 30, 60, 90, and 120, and on day 120, a high-precision soil testing instrument was directly inserted into the soil to measure in situ the physicochemical properties of the soil (including total nitrogen, total phosphorus, total potassium, and pH). Urease, dehydrogenase, and catalase activities were measured on day 120 using Solarbio enzyme activity kits (Beijing Solarbio Science & Technology Co., Ltd. (Beijing, China), urease: BC0120; dehydrogenase: BC0390; catalase: BC0100).

After processing the soil to remove impurities, the total DNA of five treatments was extracted on day 120 using the FastDNA^®^ SPIN Kit for Soil (MP Biomedicals™, Irvine, CA, USA) for high-throughput sequencing by Shanghai Majorbio Technology Co., Ltd. (Shanghai, China). Specific barcoded primers were used for PCR amplification. Sequencing was performed on the Illumina MiSeq 3000 platform. Based on the obtained representative amplicon sequencing variants (ASVs) and abundance data, the *alpha* diversity indices (Chao1, Sobs, Shannon, ACE, and Simpson) were calculated. Beta diversity between groups was evaluated via principal coordinate analysis (PCoA) using R software (version 3.3.1). Venn and Circos analyses were performed to assess microbial community composition. Prokaryotic functional annotation software (PICRUSt2) was used to predict bacterial function. Network analysis was performed to predict key microbial groups in the bacterial network under various treatment conditions, based on Spearman correlation indices.

### 2.7. Data Processing and Analysis

Data visualization was carried out using Origin 2024 software. Statistical analysis of variance was conducted using IBM SPSS Statistics (version 20.0), with statistical significance determined at *p* < 0.05. The GenBank accession numbers for the 16S rRNA gene sequences of *Cytobacillus* sp. SY-2, *Rhodococcus* sp. SY-3, *Rhodococcus* sp. SY-4, and *Rhodococcus* sp. SY-5 are PQ882138, PQ882143, PQ882221, and PQ882220, respectively. The sequencing data from the Illumina MiSeq platform has been submitted to the NCBI database under accession number PRJNA1240884.

## 3. Results

### 3.1. Identification of Petroleum-Degrading Bacterial Strains and Phylogenetic Position

**Figure 1:**
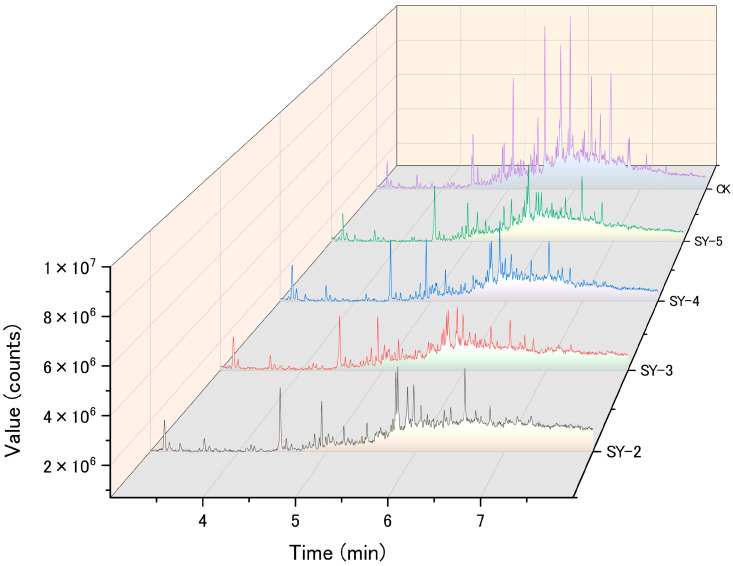
Gas chromatogram of media containing 5.0 g·L^−1^ of petroleum before (CK) and after degradation by petroleum-degrading bacteria (SY-2, SY-3, SY-4, SY-5).

Testing revealed that the petroleum used in this study was mainly composed of straight-chain alkanes, branched-chain alkanes, aromatic hydrocarbons, and non-hydrocarbon substances. The straight-chain alkanes were primarily n-tridecane and n-tetradecane, while the branched-chain alkanes were mainly heptadecane and hexacosane. Gas chromatography analysis of the petroleum degradation for each strain is shown in Figure 1. Compared to the control (CK), the four bacterial strains (SY-2, SY-3, SY-4, and SY-5) exhibited significant degradation of the main alkanes in the petroleum, with different preferences for various petroleum components.

Cells of strain SY-2 are short and rod-shaped. After culturing on solid LB medium for 3 d, colonies of 2.0–5.0 mm with smooth edges and a soft, uniform, opaque light yellow color were formed. Strains SY-3, SY-4, and SY-5 were also short, rod-shaped cells (Figure S1). After culturing on solid LB medium for 3 d, they formed uniform, smooth, small, round, opaque, white colonies of 1.0–2.0 mm in size. As summarized in Table 1, all four strains share similar profiles for common carbon source utilization and major enzyme activities. However, SY-2 uniquely possesses esterase (C4) activity, and differences are also observed in L-arginine, urea, Tween hydrolysis, and H_2_S generation, indicating a certain degree of metabolic diversity among these strains. Other physiological and biochemical characteristics are shown in Table 1. Phylogenetic analysis of the strains showed that SY-2 belongs to the genus *Cytobacillus*, while SY-3, SY-4, and SY-5 belong to the genus *Rhodococcus* on the same branch (Figure S2). Based on the analysis above, strain SY-2 was identified as a species of *Cytobacillus*, named *Cytobacillus* sp. SY-2, and strains SY-3, SY-4, and SY-5 were identified as species of *Rhodococcus*, named *Rhodococcus* sp. SY-3, *Rhodococcus* sp. SY-4, and *Rhodococcus* sp. SY-5, respectively.

### 3.2. Degradation Characteristics of the Microbial Consortium

**Figure 2:**
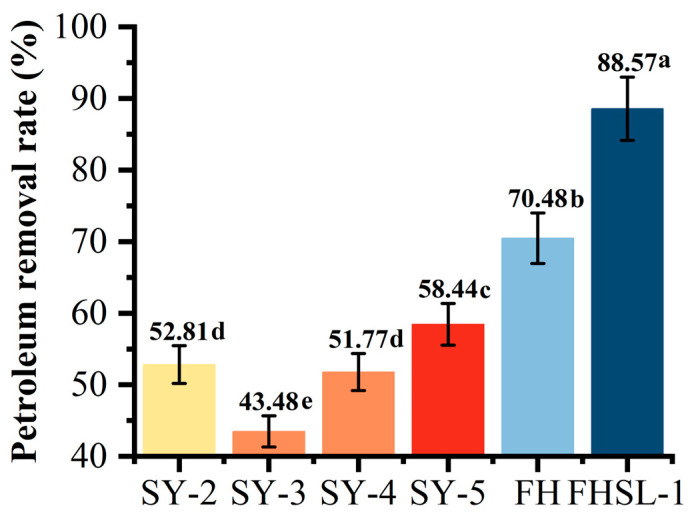
Degradation rate of petroleum by bacteria alone or mixed consortia. Different lowercase letters above the bars indicate significant differences between groups at the 0.05 level; bars sharing the same letter are not significantly different.

The degradation effects of the single strains and the microbial consortium are shown in Figure 2. The degradation rates of strains SY-2, SY-3, SY-4, and SY-5 and the microbial consortia FH and FHSL-1 were 52.8%, 43.5%, 51.8%, 58.4%, 70.5%, and 88.6%, respectively. The FHSL-1 microbial consortium exhibited the highest degradation rate (88.6%), significantly outperforming other single and mixed strains. The FH microbial consortium (70.5%) also demonstrated strong synergistic effects, indicating that the consortia effectively enhanced petroleum degradation by leveraging the strengths of the individual strains. Notably, SY-3 showed the lowest degradation rate (43.5%), suggesting limitations in its petroleum degradation capacity. Strains SY-2, SY-4, and SY-5 achieved moderate degradation rates of 52.8%, 51.8%, and 58.4%, respectively. Based on these preliminary tests, subsequent studies on immobilization and combined remediation for contaminated soil were performed using the FHSL-1 microbial consortium.

The degradation characteristics of the consortium are shown in Figures S3 and S4. The degradation rate of the microbial consortium negatively correlated with the petroleum concentration. Specifically, as the concentration of petroleum hydrocarbons increased, the degradation efficiency decreased. At 2.0 and 3.0 g·L^−1^ petroleum levels, the consortium achieved near-complete degradation within 4 d, with the residual petroleum level approaching 0 g·L^−1^. At 5.0 g·L^−1^, complete degradation was still achieved within 4 d, indicating no significant impact on the degradation efficiency. However, the degradation rate dropped to 73.1% at petroleum levels of 7.0 g·L^−1^, and further declined to 59.0% at 9.0 g·L^−1^, with a marked increase in petroleum residues, suggesting limited adaptability of the consortium under higher petroleum concentrations (Figure S3). These results suggest that higher petroleum concentrations increase metabolic stress or toxicity, reducing the degradation efficiency of the consortium. The concentration of 5.0 g·L^−1^ petroleum was identified as optimal for stable and efficient degradation, and was used in subsequent experiments to optimize the cultivation conditions. Optimal degradation was achieved at pH 7.0 (85.8%), 30 °C (84.5%), 4.0% inoculum (86.6%), and 1.0% NaCl (82.8%) (Figure S4). Extreme temperatures, deviation from neutral pH, inappropriate inoculum levels, or excessive salinity (osmotic pressure) inhibited the degradation efficiency by affecting microbial growth and metabolism. Optimizing these environmental parameters significantly improves petroleum degradation and enhances the effectiveness of petroleum pollution remediation.

### 3.3. Characterization of Immobilized Microbial Microspheres

**Figure 3:**
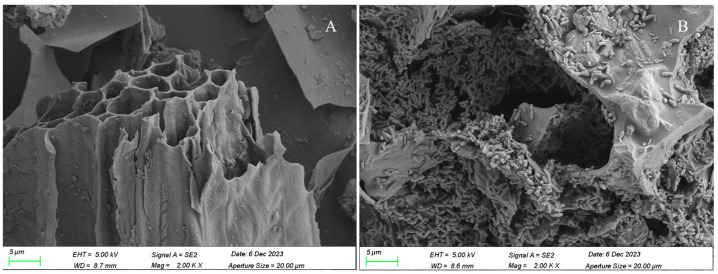
Electron microscope scanning of immobilized bacterial strains: (**A**) blank biochar; (**B**) biochar-immobilized microbial agents.

The SEM results of the biochar and adsorbed bacterial strains are shown in Figure 3. The biochar presented a porous structure, with abundant adsorption sites that could adsorb a large amount of bacteria. Figure S5 shows that after embedding with SA, the biochar-adsorbed bacteria formed microspheres consisting of regular small particles, black in color, with a diameter of approximately 0.38 cm and a mass of approximately 0.03 g per sphere. This indicates that the adsorption-embedding immobilization process was successful.

The effects of biochar, SA, and CaCl_2_ concentrations on the petroleum degradation rate of the immobilized microspheres are shown in Figure S6 and Table S1. When the SA, biochar, and CaCl_2_ concentrations were 40 g·L^−1^, 0.75 g·L^−1^, and 40 g·L^−1^, respectively, the microspheres achieved the highest petroleum degradation rates of 82.7%, 88.6%, and 78.6%, respectively. Using the biochar (C), SA (A), and CaCl_2_ (B) concentrations as independent variables and the petroleum degradation rate (Y) as the response value, a Box–Behnken design was used to optimize the immobilization process (Table 2). The experimental results were regressed using Design-Expert 13.0 software to obtain the regression equation (Table S2 and Table 3). The regression equation of the optimal preparation conditions was Y = 95.42 − 3.01A − 6.01B − 3.01C − 1.62AB − 0.525AC − 0.325BC − 10.55A^2^ − 16.91B^2^ − 15.06C^2^, which showed a good fit (R^2^ = 0.9920, R^2^Adj = 0.9818, *p* < 0.0001). Significance analysis revealed that the factors influencing the petroleum degradation rate (Y) were ordered as follows: biochar content (C) > SA content (A) > CaCl_2_ concentration (B). After combining the Box–Behnken design and regression analysis, the optimal immobilization conditions were 0.69 g·L^−1^ of biochar, 42.5 g·L^−1^ of SA, and 36.5 g·L^−1^ of CaCl_2_. To improve practical feasibility, these factors were adjusted to 0.75 g·L^−1^ of biochar, 40 g·L^−1^ of SA, and 40 g·L^−1^ of CaCl_2_. After conducting three parallel experiments under these conditions, the average petroleum degradation rate was 97.1%, which is consistent with the theoretical value of 97.6%.

**Figure 4:**
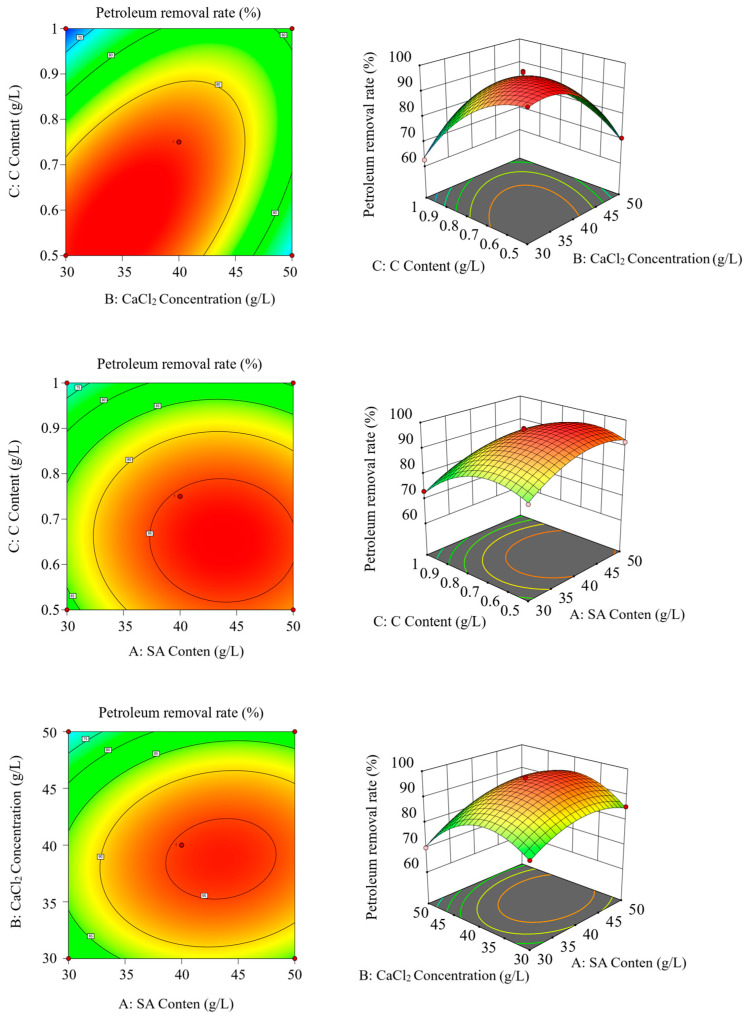
Response surface and contour plots showing the effect of microsphere preparation factors on petroleum removal rate.

The response surface and contour plots showing the influence of different factors on the petroleum degradation rate are shown in Figure 4. These plots visually reflect how interactions between factors affect the response. The steeper the surface and the denser the contour lines, the more significant the effect. The most significant interaction occurred between the CaCl_2_ and the biochar concentrations, followed by that between the SA and the CaCl_2_ concentrations. The interaction between the SA and the biochar contents had little impact. The optimization scheme significantly improved the petroleum degradation efficiency of the microspheres, providing a reliable theoretical basis for subsequent application of the immobilized microbial consortium.

### 3.4. Screening of Petroleum Remediation Plants

**Figure 5:**
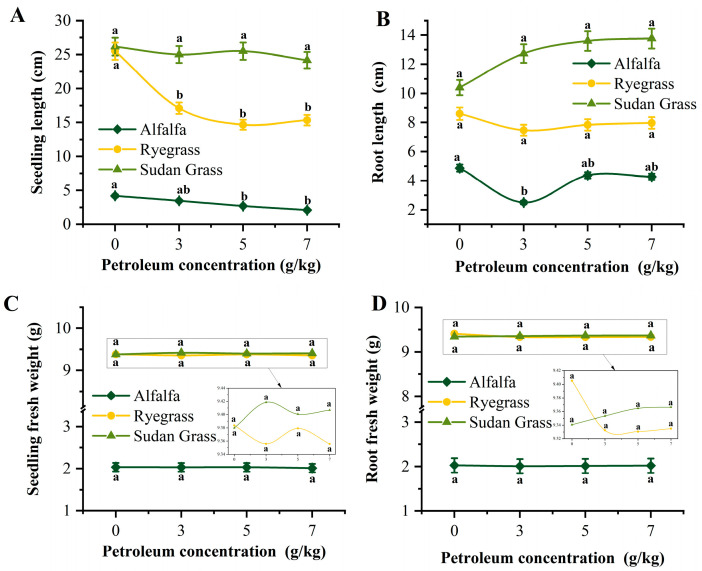
Growth of tested plants in petroleum-contaminated soil: (**A**) seedling length; (**B**) root length; (**C**) seedling fresh weight; (**D**) root fresh weight of alfalfa, ryegrass and Sudan grass under different petroleum concentrations. For each plant species, groups with different lowercase letters at different petroleum concentrations are significantly different (*p* < 0.05, *n* = 3).

Germination rates of the three tested plants grown in petroleum-contaminated soil are shown in Figure S7. Under non-contaminated conditions, the germination rates of the three plants were above 90%, which confirms good seed quality. Sudan grass showed no significant change in germination rate (90.0–96.7%) under the different petroleum contamination concentrations, while alfalfa and ryegrass showed suppressed germination rates as the contaminant concentration increased (43.3–95.0% and 76.7–91.7%, respectively). Sudan grass grew best in petroleum-contaminated soil, with stable seedling height and root length, as well as enhanced root growth (Figure 5A,B), demonstrating strong tolerance. Ryegrass exhibited a slight decrease in seedling height and suppressed root development at high petroleum contamination concentrations (Figure 5C,D). Alfalfa performed poorly under the contaminated conditions, with significant inhibition of both seedling height and root length (Figure 5A,B). Overall, Sudan grass exhibited the best adaptability to the petroleum-contaminated soil, with strong soil improvement potential, making it the preferred plant for further research. Additionally, at 7.0 g·kg^−1^ petroleum contamination, Sudan grass had a total nitrogen (TN) content of 42 mg·kg^−1^, while that of alfalfa decreased to 39.33 mg·kg^−1^ (Figure S8A). The total phosphorus (TP) content of Sudan grass was 57.33 mg·kg^−1^, whereas that of both alfalfa and ryegrass showed a significant decline (Figure S8B). The total potassium (TK) content of Sudan grass was 130.33 mg·kg^−1^, while that of alfalfa and ryegrass decreased to 120 and 104 mg·kg^−1^, respectively (Figure S8C). Compared to ryegrass and alfalfa at the same petroleum concentration, Sudan grass exhibited the highest TN, TP, and TK contents in the soil, indicating that Sudan grass can thrive in petroleum-contaminated soil, making it a promising candidate for subsequent combined remediation.

### 3.5. Combined Immobilized Microbial Consortium and Sudan Grass for Enhanced Remediation of Petroleum-Contaminated Soil

#### 3.5.1. Petroleum Degradation Effect of Contaminated Soil

**Figure 6:**
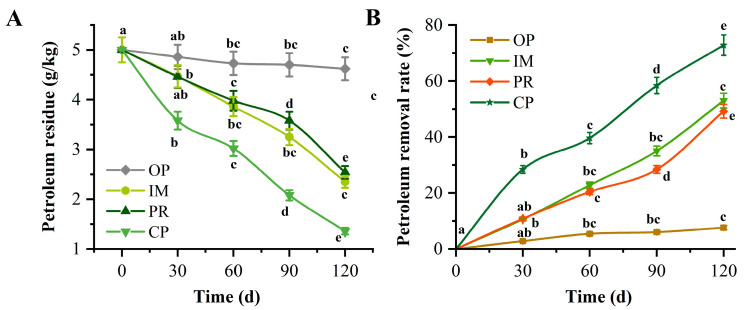
Removal of petroleum in soil under different treatments. (**A**): Petroleum residue (g·kg^−1^); (**B**): Petroleum removal rate (%) under OP, IM, PR, and CP treatments. For each treatment, groups with different lowercase letters at different time points are significantly different (*p* < 0.05, *n* = 3).

Petroleum degradation under different treatment conditions varied significantly (Figure 6). After 120 d, the remaining petroleum concentration in the OP treatment (without biological remediation) was 4.62 g·kg^−1^ (Figure 6A), registering a degradation rate of 7.6%. The petroleum concentration in the IM (immobilized microorganisms) and PR (plant remediation) treatments was 2.35 and 2.54 g·kg^−1^, respectively, representing degradation rates of 53.0% and 49.2% (Figure 6B), respectively. The CP treatment (combined remediation) performed the best, reducing the remaining petroleum concentration to 1.36 g·kg^−1^, which represents a degradation rate of 72.8%. This indicates that in terms of remediation efficiency, the combined immobilized microbial consortium and Sudan grass is most effective in removing petroleum from the soil.

#### 3.5.2. Soil Physicochemical Properties and Enzyme Activity

The soil physicochemical properties under different treatments are shown in Figure S9. Petroleum contamination significantly reduced soil fertility and pH. The OP treatment showed TN, TP, and TK contents of 23.3, 31.0, and 106.7 mg·kg^−1^, respectively, with a pH of 5.7. The IM and PR treatments significantly improved soil fertility, with the TN, TP, and TK contents ranging from 41.7–44.7, 58.0–67.7, and 157.7–155.3 mg·kg^−1^ (Figure S9A), respectively, and pH increasing to 6.0–6.1 (Figure S9B). The CP treatment showed the most significant improvement, with TN, TP, and TK contents reaching 60.0, 83.7, and 192.0 mg·kg^−1^, respectively, and pH increasing to 6.4. During the process of petroleum-contaminated soil remediation, the soil enzyme activity is a critical indicator of microbial activity and remediation effectiveness. Urease is related to the soil nitrogen cycle, as it hydrolyzes carbon–nitrogen bonds in organic matter, thereby promoting the conversion of organic nitrogen into available nitrogen, which supports microbial growth in the soil. As key enzymes, dehydrogenase and catalase play an essential role in petroleum hydrocarbon degradation and thus, in the reduction of soil toxicity. The effects of different treatments on soil enzyme activities are shown in Figure S10. Petroleum contamination significantly reduced the activity of urease (Figure S10A), dehydrogenase (Figure S10B), and catalase (Figure S10C). All remediation treatments showed an increase in enzyme activity, with the combined remediation treatment reaching 481.79, 10.67, and 19.67 U·g^−1^ for urease, dehydrogenase, and catalase, respectively, representing an increase of 164.5%, 152.8%, and 172.4%, respectively, compared to results for the petroleum-contaminated control. This indicates that combined remediation can significantly enhance soil nutrients, improve enzyme activity, and adjust pH.

#### 3.5.3. Bacterial Community Analysis of Petroleum-Contaminated Soil Remediation

*Alpha* diversity analysis (Table 4) showed that the species richness (Sobs, ACE, Chao1 indexes) and diversity (Shannon and Simpson indexes) were significantly lower in the OP treatment (no biological remediation) compared to in the control (CK) (*p* < 0.05). The Sobs resultof CK was 3032, while that of OP was 2371, indicating that contamination had a significant inhibitory effect on the microbial community. Compared to the petroleum-contaminated treatments, the species richness and diversity were increased in several remediation treatments, with CP (combined remediation) showing a significantly better Simpson index score compared to that of OP (*p* < 0.05). This suggests that combined remediation using the immobilized microbial consortium and Sudan grass could alleviate stress on the microbial community, which affects richness and diversity, caused by petroleum contamination, thereby laying a solid foundation for soil ecological restoration.

**Figure 7:**
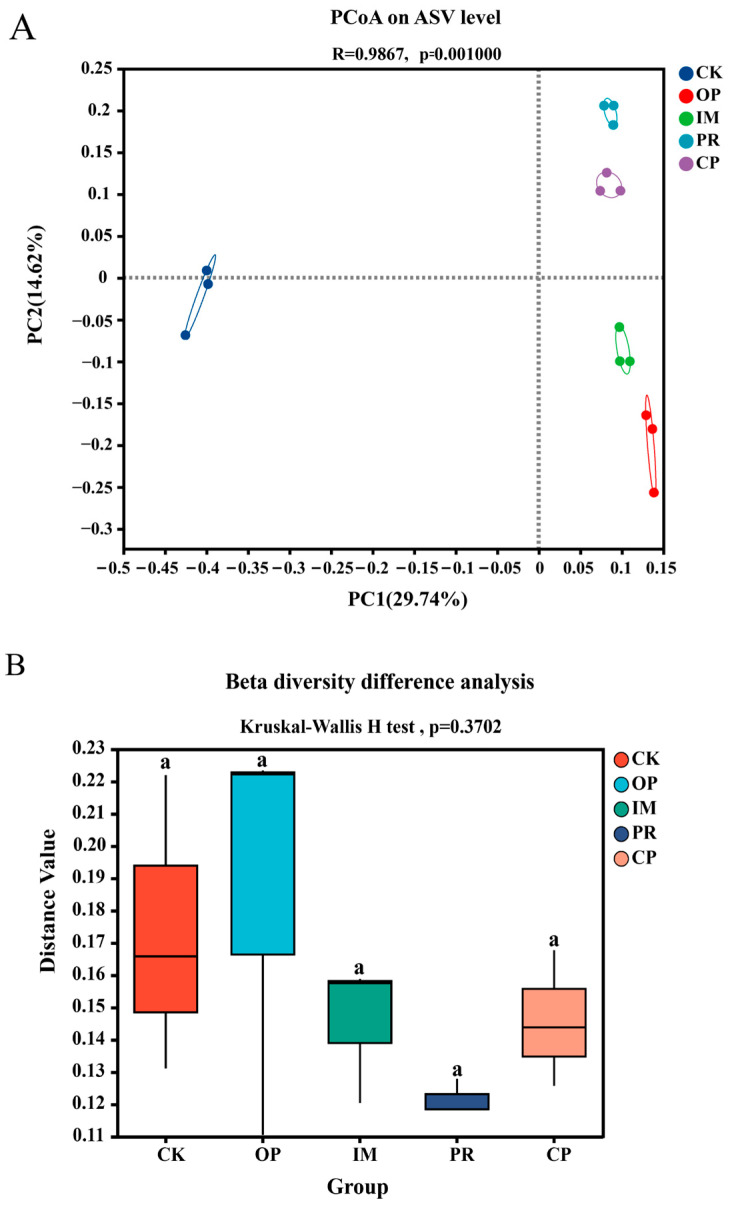
Intergroup differences analysis of beta diversity among different soil samples: (**A**) principal coordinates analysis (PCoA) based on ASV level showing the ordination of microbial communities among different treatments; (**B**) beta diversity difference analysis among groups. Groups with the same lowercase letter are not significantly different at *p* < 0.05 (Kruskal–Wallis H test).

To further assess the impact of different treatments on the soil microbial community, *beta* diversity analysis was conducted and visualized using PCoA. The PCoA shows that the microbial community structure significantly changed in the different treatments, with clear clustering of groups on the coordinate axes (Figure 7A). The CK treatment was distinctly separated from other treatments, indicating that petroleum contamination significantly altered the soil microbial community structure (Figure 7B). The distinct separation between the OP and CK treatments indicates that the pollutants exerted strong disturbance on the microbial composition, leading to deep changes in the community structure. The PCoA shows that although different remediation treatments showed varying degrees of community recovery, none recovered to the pre-contaminated state.

**Figure 8:**
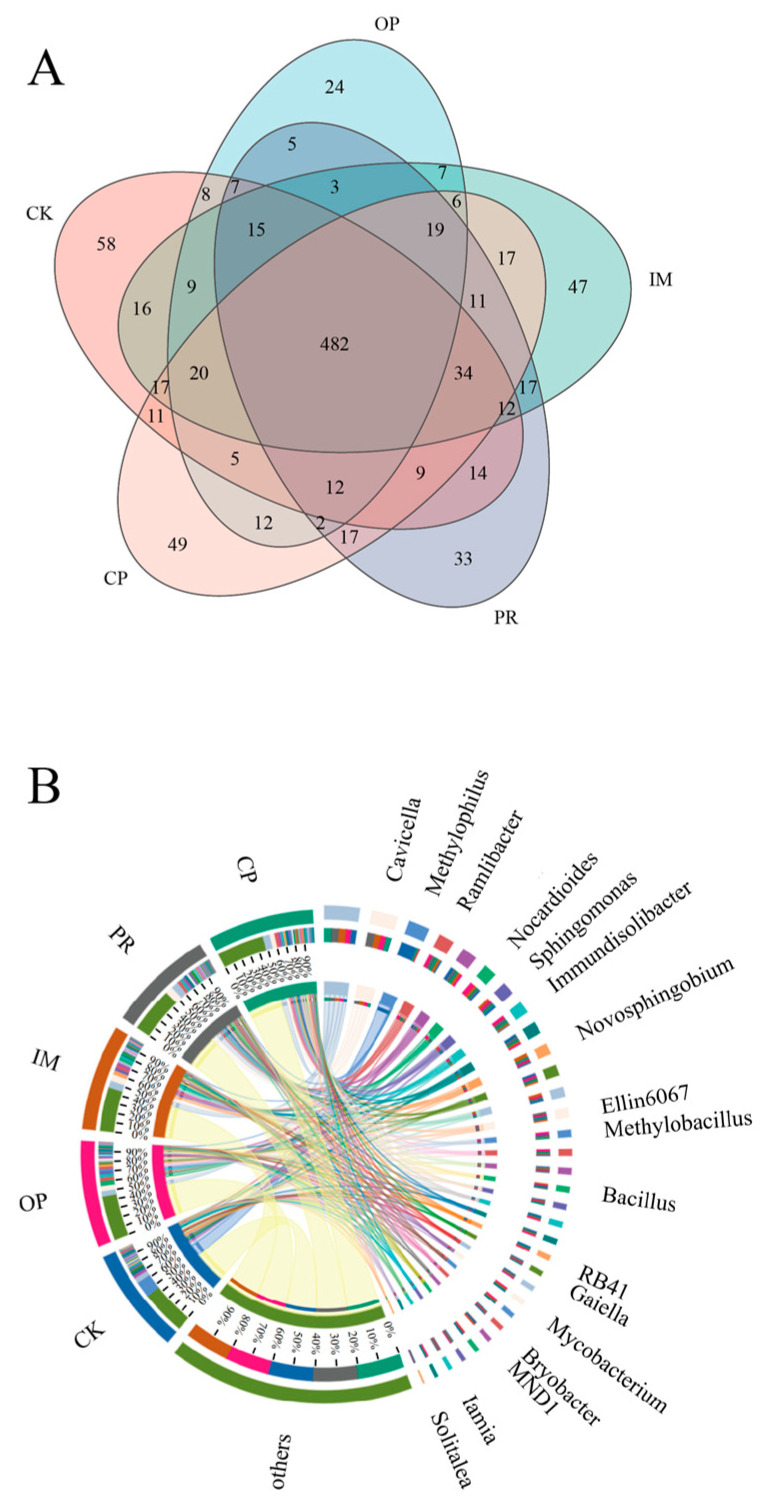
Venn (**A**) and Circos (**B**) analysis of different soil samples at the genus level.

Venn and Circos analyses (Figure 8) revealed significant differences in microbial community composition among different treatments. The CK treatment contained 729 genera, with 58 being unique. *Methylophilus* showed the highest relative abundance at approximately 16.65%, while *Nocardioides* and *Sphingomonas* accounted for about 1.21% and 1.91%, respectively. In the OP treatment, the total number of genera decreased to 636, with only 24 being unique. The dominant genera were *Cavicella* (5.16%) and *Nocardioides* (4.91%). *Methylophilus* significantly decreased, and other genera showed lower relative abundances compared to that of CK. In the remediation treatments, IM contained 732 genera (47 unique), with *Cavicella* (6.73%) and *Novosphingobium* (4.07%) being the dominant types; PR contained 685 genera (33 unique), with *Cavicella* (7.65%) and *Ramlibacter* (3.39%) as dominant; while CP contained 723 genera, with 49 being unique (the highest number of unique genera in the remediation treatments), and several dominant genera showing higher relative abundances relative to those of the OP treatment. *Cavicella* (4.74%) and *Ramlibacter* (3.20%) displayed higher relative abundances in the CP treatment, and some genera, such as *Solitalea*, were detected at a higher abundance in the CP treatment than in the other treatments. All remediation treatments suggested that petroleum contamination led to a significant decrease in the number of total and unique genera, with combined remediation (CP) performing the best in regards to restoring unique genera and adjusting the relative abundance of the dominant genera.

PICRUSt2 was used to predict the bacterial community function for different treatments (Figure S11), identifying 22 secondary metabolic pathways. The CK treatment (control) maintained stable functional properties, reflecting a healthy soil microbial ecosystem. Petroleum contamination (OP) enhanced functions related to carbohydrate transport and metabolism, as well as energy production and conversion, reflecting the microbial stress response to pollution. Among the remediation measures, the IM (immobilized microbial consortium) and PR (plant remediation) treatments showed improvements in both defense mechanisms and cell cycle regulation, but metabolic recovery was still lower than that of the CK treatment. CP (combined remediation) performed the best, with signal transduction mechanisms reaching the highest level and significant optimization of core functions (such as amino acid transport and metabolism, as well as energy production and conversion), approaching or exceeding those of CK. Overall, the CK treatment maintained healthy function; OP exhibited functional remodeling under pollution stress; the IM and PR treatments showed limited recovery; and CP achieved optimal metabolic recovery through combined remediation.

**Figure 9:**
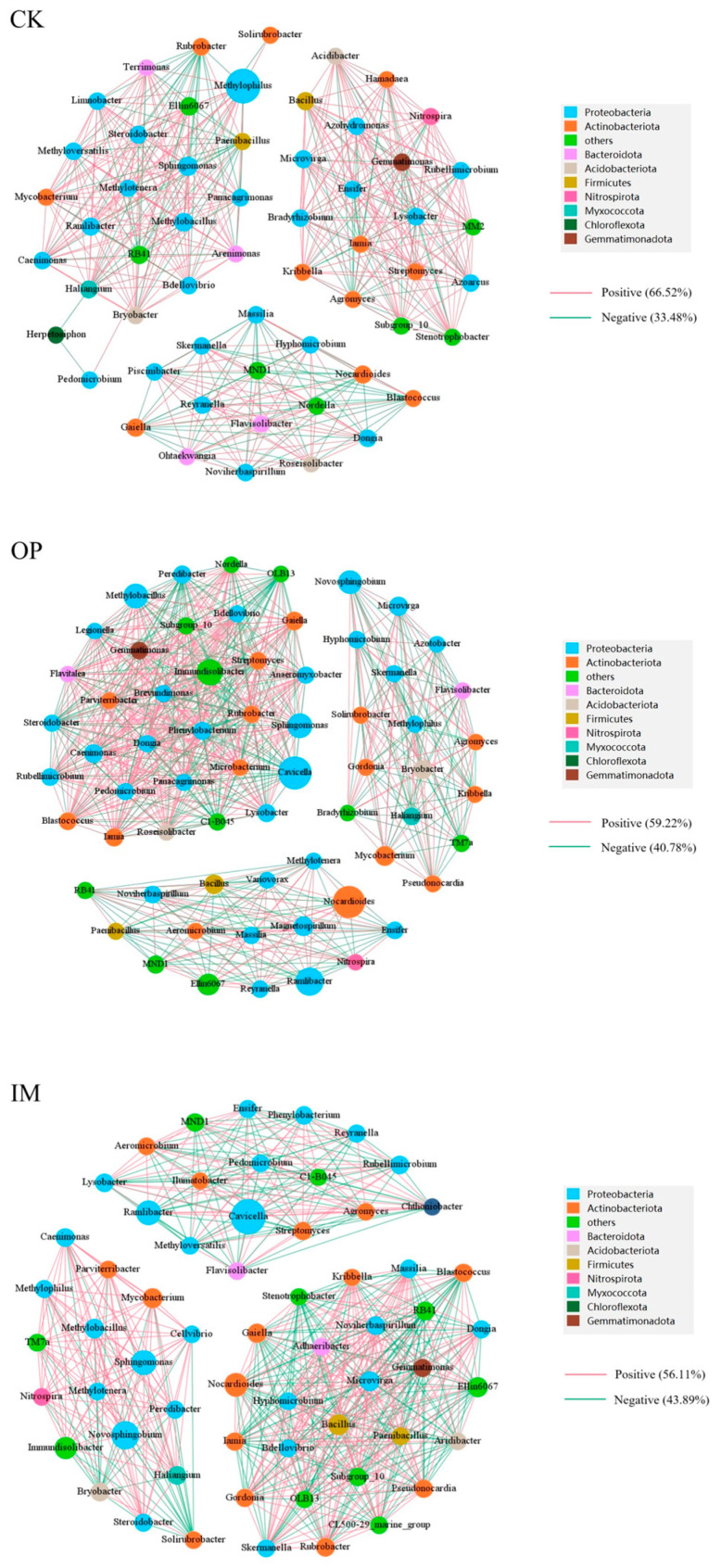

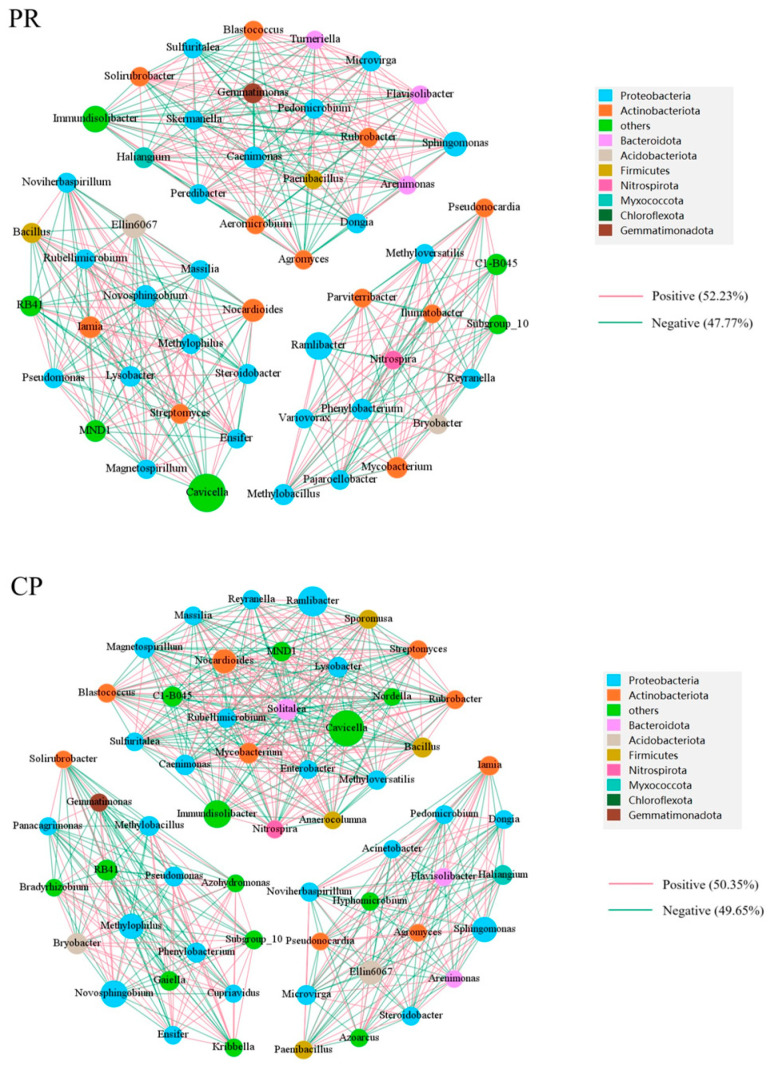
Bacterial network analysis of different soil samples.

Based on the Spearman correlation (*p* < 0.05, correlation threshold 0.6, maximum species number 100), a bacterial co-occurrence network was constructed (Figure 9). The different remediation strategies significantly affected the microbial interaction patterns in the soil. CK (uncontaminated control) exhibited a tightly connected network totaling 1497 connections, of which 808 were positive and 689 were negative. The main genera in the network belonged to the phyla Proteobacteria, Actinobacteriota, Bacteroidota, and Firmicutes. *Arenimonas* and *Steroidobacter* showed the highest connection numbers of 36 and 35, respectively. The OP treatment (petroleum contamination) showed an increased number of connections (1717; 923 positive, 794 negative). *Lysobacter* and *Panacagrimonas* had the highest connection numbers of 44 and 43, respectively. This indicates that petroleum contamination enhanced bacterial competition, reducing community stability. The IM treatment (immobilized microbial consortium) displayed 1706 connections (950 positive, 756 negative). The PR treatment (plant remediation) revealed 1557 connections (780 positive, 777 negative), with those of *Lysobacter* and *Noviherbaspirillum* being the highest (33 and 32, respectively). The CP treatment (combined remediation) displayed 1654 connections (821 positive, 833 negative), with *Cavicella* and *Immundisolibacter* having the highest connections (39 and 38, respectively). The relationships between Proteobacteria and Actinobacteriota increased, approaching or exceeding those of the CK treatment. Overall, combined remediation not only enriched the potential petroleum-degrading genera (such as *Pseudomonas* [38] and *Bacillus* [39]) but also optimized the microbial network stability (*Sphingomonas* [40]), thereby enhancing the recovery capacity of the soil ecosystem.

## 4. Discussion

Research on the microbial remediation of petroleum-contaminated soil has reached a bottleneck. Although petroleum-degrading strains have been isolated in recent years from genera such as *Bacillus* [41], *Enterobacter* [42], and *Acinetobacter* [43], there is still limited availability of efficient strains. In this study, four petroleum-degrading strains were isolated from petroleum-contaminated soil, with the highest single-strain degradation rate reaching 58.4%. These isolates belong to the genera *Cytobacillus* and *Rhodococcus*, thus enriching the resource bank of petroleum-degrading microbial strains and offering new possibilities for bioremediation. Furthermore, as single-strain degradation of petroleum is limited, consortium-based remediation has become a focus of research. In this study, the four isolated strains showed different preferences for petroleum components, indicating the potential for synergistic degradation. Indeed, the degradation rate of the constructed four-strain microbial consortium increased to 70.5%, consistent with the results of previous research [44]. In addition, biosurfactants can enhance the solubility of hydrophobic petroleum hydrocarbons, boosting their microbial degradation capabilities [45]. Using *Pseudomonas aeruginosa* for bioaugmentation and rhamnolipid biosurfactant production, Lopes et al. achieved a total degradation of 90% in 181 d when treating lubricating oil-contaminated soil [46]. Additionally, Smita et al. found that rhamnolipids promote the degradation of PAHs [47]. In this study, creating a consortium by combining the rhamnolipid-producing strain *Pseudomonas aeruginosa* SL-1 with the four petroleum-degrading strains increased the degradation rate from 70.5% to 88.6%, consistent with previous findings and providing a new technique for bioaugmentation-based remediation. However, the addition of exogenous strains to contaminated environments often faces challenges such as low strain survival and an unstable degradation efficiency. Immobilization can stabilize the microbial community, prevent strain loss, and enhance degradation persistence. Sonsuphab et al. showed that the biochar immobilization of microorganisms reached a 97.0% removal efficiency for triclocarban contamination [48]. Wu et al. developed immobilized fungi microspheres using CaCl_2_ and SA, achieving a 94.7% removal rate of 2,4,6-trichlorophenol under optimal conditions [49]. In this study, immobilizing the microbial consortium on SA–biochar successfully addressed the mass transfer limitations of SA and the desorption issue of biochar, effectively improving bacterial activity and the mass transfer efficiency. It should be noted that although *Pseudomonas aeruginosa* SL-1 was introduced in this study to enable the in situ biosynthesis of rhamnolipids and a significant enhancement in hydrocarbon degradation was observed, the actual production of rhamnolipids in soil was not quantitatively determined, representing a limitation of this work. Given that rhamnolipids can be adsorbed onto soil organic matter and mineral surfaces, their freely available concentrations and effective activity may fluctuate under natural conditions. The absence of production data restricts a more precise understanding of their dynamic behavior and functional mechanism. Future studies will incorporate LC-MS/MS and related techniques to quantify rhamnolipid levels in soil and elucidate their spatiotemporal distribution, aiming to clarify their role in hydrocarbon desorption, bioavailability enhancement, and overall remediation efficiency.

For petroleum-contaminated soil, microorganism- and plant-based remediation each express their advantages. However, the complex composition, high toxicity, and long degradation cycle of petroleum renders single remediation methods limited and inefficient. Combined microorganism–plant-based remediation could complement the advantages of each method and improve the overall degradation efficiency [15]. Zhao et al. demonstrated that, in farmland soils contaminated with PAHs and Cd, the combination of microbial degradation/mineralization and plant absorption, along with specific soil amendments, could increase the remediation efficiency up to 93.8% [50]. Wu et al. used the cooperative function of *Phragmites australis* and fungi to remediate petroleum-contaminated soil, with the petroleum degradation rate of the plants significantly increasing after fungal inoculation [51]. In this study, the combined immobilized microorganisms and Sudan grass achieved a petroleum degradation rate of 72.8% after 120 d of pot cultivation in petroleum-contaminated soil, significantly improving soil nutrients, as well as the urease, dehydrogenase, and catalase activities, and effectively restoring the soil ecology.

Bacterial community analysis of the petroleum-contaminated soil revealed significant differences among treatments. Petroleum contamination significantly reduced the *alpha* diversity, altered the *beta* diversity, reduced the microbial diversity (as demonstrated in the Venn analysis), disrupted the genus-level bacterial community structure, changed the community function, enhanced bacterial competition, and decreased the co-occurrence network stability. Although the immobilized microbial remediation and plant remediation both showed certain beneficial effects, the combined remediation exhibited the best performance. Combined remediation increased the *alpha* diversity, partially restored the *beta* diversity, and promoted the enrichment of unique genera. Notably, the CK, OP, IM, PR, and CP treatments registered 729, 636, 732, 685, and 723 core genera, respectively, in the Venn analysis, with CP displaying 49 unique genera, the most core genera, and the strongest bacterial community regulation and enrichment effect. Thus, the CP treatment optimized the genus-level bacterial community structure, achieved the best community functional metabolic recovery, and demonstrated optimal microbial co-occurrence network stability. In the immobilized microbial consortium, *Cytobacillus* (SY-2) and *Rhodococcus* (SY-3, SY-4, SY-5) were not detected within the later community data, which may be related to their “short-term driving” strategy. Due to their high petroleum hydrocarbon degradation efficiency, these genera rapidly released metabolic products (such as organic acids and surfactants) during the early remediation stage, which facilitated the recovery of native microbial populations [52]. However, as the petroleum hydrocarbon concentration decreased, the carbon sources on which these exogenous strains rely diminished, reducing their competitiveness. Eventually, they were replaced by native microorganisms (such as *Ramlibacter*, *Nocardioides,* and *Sphingomonas*) with stronger ecological niche adaptability. Ecological niche competition is another important factor. *Cytobacillus* and *Rhodococcus* may provide a favorable habitat for native bacteria during the early remediation stages, but in the later stages, native bacteria gradually replace the exogenous strains as the soil bacterial community is rebuilt, leading to a decline in or disappearance of the exogenous strains [53]. Furthermore, although immobilized carriers can provide a stable growth environment over the short term, the survival pressure on exogenous strains increases as pollutants are degraded, which may lead to insufficient adaptability over the long term [54]. In particular, the plant root exudates of the CP treatment might further promote the dominance of native bacteria, accelerating the decline of exogenous strains. In summary, the petroleum degradation effect of *Cytobacillus* and *Rhodococcus* is mainly reflected during the early remediation phase. Later, due to carbon source depletion, ecological niche competition, and environmental adaptability, these strains are replaced by native microorganisms. This transient but crucial effect not only accelerates pollutant degradation, but also promotes the enrichment of native microorganisms by releasing metabolic products and shaping the ecological niche, thereby providing important support for the reconstruction of the soil microbial community. Although the immobilized microbial consortium was not enriched in the final community structure, its “behind-the-scenes” effect ensured efficient operation of the remediation system. This study applied the combined immobilized microbial consortium–plant remediation strategy to achieve a significant increase in petroleum degradation, ultimately optimizing the soil bacterial community and function. Future research could incorporate plant enzymatic activity measurements as experimental controls to more accurately quantify the specific contribution of plants to pollutant degradation or accumulation within plant–microbe systems. In addition, further in-depth investigation into the molecular and biochemical interactions between plants and microbes—such as those involving root exudates, signaling molecules, and plant metabolites under stress—will help to elucidate the synergistic mechanisms underlying efficient bioremediation.

## 5. Conclusions

This study constructed a petroleum-degrading microbial consortium system consisting of *Cytobacillus* sp. SY-2, *Rhodococcus* sp. SY-3, *Rhodococcus* sp. SY-4, *Rhodococcus* sp. SY-5, and *Pseudomonas aeruginosa* SL-1. After using response surface methodology to optimize the immobilization conditions (0.75 g·L^−1^ of biochar, 40 g·L^−1^ of SA, 40 g·L^−1^ of CaCl_2_), the petroleum (5 g·L^−1^) degradation rate of the immobilized microspheres reached 97.1% within 72 h. When this immobilized microbial consortium was applied in combination with Sudan grass to treat petroleum-contaminated soil, the petroleum degradation rate reached 72.8% after 120 d, which was significantly higher than that for the treatments using only the immobilized microbial consortium (53.0%) or plant remediation (49.2%). Additionally, soil pH, TN, TP, TK, as well as urease, dehydrogenase, and catalase activities, were significantly improved. Moreover, combined remediation significantly increased the diversity and richness of the soil bacterial community; optimized the structure, function, and network composition of the soil bacterial community; promoted the recovery of the microbial community in the contaminated soil; and enriched specific genera, thereby enhancing the stability and recovery capacity of the soil ecosystem. This study provides an effective method and theoretical basis for enhanced remediation of petroleum-contaminated soil. The combined immobilized microbial consortium + plant remediation strategy shows good application prospects and may provide new ideas and practical support for remediating petroleum contamination in soils.

## Figures and Tables

**Table 1:** Physiological and biochemical characteristics of strains.

Indicators	SY-2	SY-3	SY-4	SY-5	Indicators	SY-2	SY-3	SY-4	SY-5
D-Ribose	+	−	−	−	D-Glucose	+	−	−	−
Sucrose	+	−	+	−	L-Arginine	−	+	+	+
Maltose	+	−	−	−	Urea	−	+	+	+
DL-Lactate	+	+	+	+	Tween 40 hydrolysis	+	+	+	+
L-Alanine	+	−	−	+	H_2_S generation	+	−	−	−
Glycogen	+	−	−	−	Tween 60 hydrolysis	+	−	−	+
L-Serine	+	−	−	−	Catalase	−	−	−	−
Mannitol	+	+	+	+	α-Glucosidase	−	−	−	−
Esterase (C4)	+	−	−	−	Oxidase	+	+	+	+

Note: “+”: positive; “−”: negative.

**Table 2:** Experimental factors and levels.

Level	Factor		
	A: SA Content (g·L^−1^)	B: CaCl_2_ Concentration (g·L^−1^)	C: C Content (g·L^−1^)
−1	30	30	0.5
0	40	40	0.75
1	50	50	1

**Table 3:** Results of response surface method.

Trial Number	Factor			Petroleum Removal Rate (%)
	A: SA Content (g·L^−1^)	B: CaCl_2_ Concentration (g·L^−1^)	C: C Content (g·L^−1^)	
1	30	30	0.75	80.1
2	50	30	0.75	85.3
3	30	50	0.75	70.0
4	50	50	0.75	81.7
5	30	40	0.5	82.7
6	50	40	0.5	91.6
7	30	40	1	73.1
8	50	40	1	79.9
9	40	30	0.5	96.7
10	40	50	0.5	70.4
11	40	30	1	62.6
12	40	50	1	77.7
13	40	40	0.75	94.5
14	40	40	0.75	93.5
15	40	40	0.75	94.9
16	40	40	0.75	97.1
17	40	40	0.75	96.9

**Table 4:** *Alpha* diversity index analysis of bacterial communities in different soil samples.

Treatment	Sobs	ACE	Chao1	Shannon	Simpson
CK	3032 ± 437 a	3106 ± 528 a	3076 ± 497 a	6.47 ± 0.28 a	0.0309 ± 0.0140 a
OP	2371 ± 154 b	2385 ± 162 b	2374 ± 156 b	6.61 ± 0.06 a	0.0060 ± 0.0016 b
IM	2656 ± 112 ab	2704 ± 129 ab	2685 ± 128 ab	6.73 ± 0.07 a	0.0070 ± 0.0009 b
PR	2648 ± 135 ab	2674 ± 131 ab	2654 ± 132 ab	6.76 ± 0.10 a	0.0064 ± 0.0013 b
CP	2595 ± 308 ab	2595 ± 308 ab	2612 ± 332 ab	6.69 ± 0.02 a	0.0071 ± 0.0008 b

Note: Different lowercase letters indicate significant differences between groups (*p* < 0.05).

## Data Availability

Data will be made available upon request.

## Supplementary Materials

The following supporting information can be downloaded at: https://www.mdpi.com/article/10.3390/toxics13070599/s1, Table S1. The effects of biochar, sodium alginate, and CaCl_2_ concentrations on the removal of petroleum by immobilized microspheres; Table S2. Analysis of variance results; Figure S1. Microscopic view of petroleum-degrading strains; Figure S2. Neighbor-joining tree of strains based on the 16S rRNA gene; Figure S3. Effect of initial petroleum concentration on degradation by the microbial consortium; Figure S4. Effects of different influencing factors on the degradation of the petroleum-degrading microbial consortium; Figure S5. Sodium alginate–biochar microspheres; Figure S6. The effects of biochar, sodium alginate, and CaCl_2_ concentrations on the removal of petroleum by immobilized microspheres; Figure S7. Germination rate of three tested plants in petroleum-contaminated soil; Figure S8. Physicochemical properties of tested plants in petroleum-contaminated soil; Figure S9. Physicochemical properties of soil under different treatments; Figure S10. Effects of different treatments on soil enzyme activity; Figure S11. Prediction of bacterial function in different soil samples.
