# Supplementary material for: Investigation into the Enhancement Effects of Combined Bioremediation of Petroleum-Contaminated Soil Utilizing Immobilized Microbial Consortium and Sudan Grass
Source: Toxics. 2025 Jul 16;13(7):599. doi: 10.3390/toxics13070599 (PMC12299197; doi:10.3390/toxics13070599)
Supplement: Supplementary file 1 [file toxics-13-00599-s001.zip › toxics-3686991-supplementary.pdf]

Supplementary materials

Investigation into the Enhancement Effects of Combined Bioremediation of Petroleum-Contaminated Soil Utilizing Immobilized Microbial Consortium and Sudan Grass

Tie-Jun Wang [1,†], Zi-Yue Ding [2,†], Zi-Wei Hua [2], Zi-Wang Yuan [2], Qiu-Hong Niu [2,*] and Hao Zhang [2,*]

[1] Henan Province Engineering Technology Research Center of Animal Disease Control and Prevention, Nanyang Vocational College of Agriculture, Nanyang 473000, China; tie20062002@163.com

[2] College of Life Science, Research Center of Henan Provincial Agricultural Biomass Resource Engineering and Technology, Nanyang Normal University, Nanyang 473061, China; 15328331055@163.com (Z.-Y.D.); 15239943441@163.com (Z.-W.H.); ziwang0329@outlook.com (Z.-W.Y.)

* Correspondence: qiuhongniu723@163.com (Q.-H.N.); zhanghao660@nynu.edu.cn (H.Z.)

† These authors contributed equally to this work.

Table S1 The effects of biochar, sodium alginate, and $CaCl_2$ concentration on the removal of petroleum by immobilized microspheres.

SA Content (g·L^{-1})	$Cacl_2$ Concentration (g·L^{-1})	C Content (g·L^{-1})	Petroleum removal rate (%)
40	40	0.5	75.3
40	40	0.75	88.6
40	40	1	80.9
40	40	1.25	78.5
40	40	1.5	76.5
20	40	1	75.3
30	40	1	79.6
40	40	1	82.7
50	40	1	78.6
60	40	1	76.6
40	20	1	76.3
40	30	1	77.6
40	40	1	78.6
40	50	1	78.2
40	60	1	77.5

Table S2 Analysis of variance results

Source	Sum of Squares	df	Mean Square	F-value	p-value	Significance
Model	1904.45	9	211.61	97.00	< 0.0001	**
A: SA Conten	132.85	1	132.85	60.90	0.0001	**
B: $Cacl_2$ Concentration	77.50	1	77.50	35.53	0.0006	**
C: C Content ($g \cdot L^{-1}$)	289.20	1	289.20	132.57	< 0.0001	**
AB	10.56	1	10.56	4.84	0.0637	
AC	1.10	1	1.10	0.5054	0.5001	
BC	428.49	1	428.49	196.42	< 0.0001	**
A^2	131.34	1	131.34	60.20	0.0001	**
B^2	469.53	1	469.53	215.23	< 0.0001	**
C^2	270.15	1	270.15	123.84	< 0.0001	**
Residual	15.27	7	2.18			
Lack of Fit	4.74	3	1.58	0.6006	0.6479	
Pure Error	10.53	4	2.63			
Cor Total	1919.72	16				

Note: * indicates a significant difference (*P*<0.05); ** indicates a very significant difference (*P*<0.01).

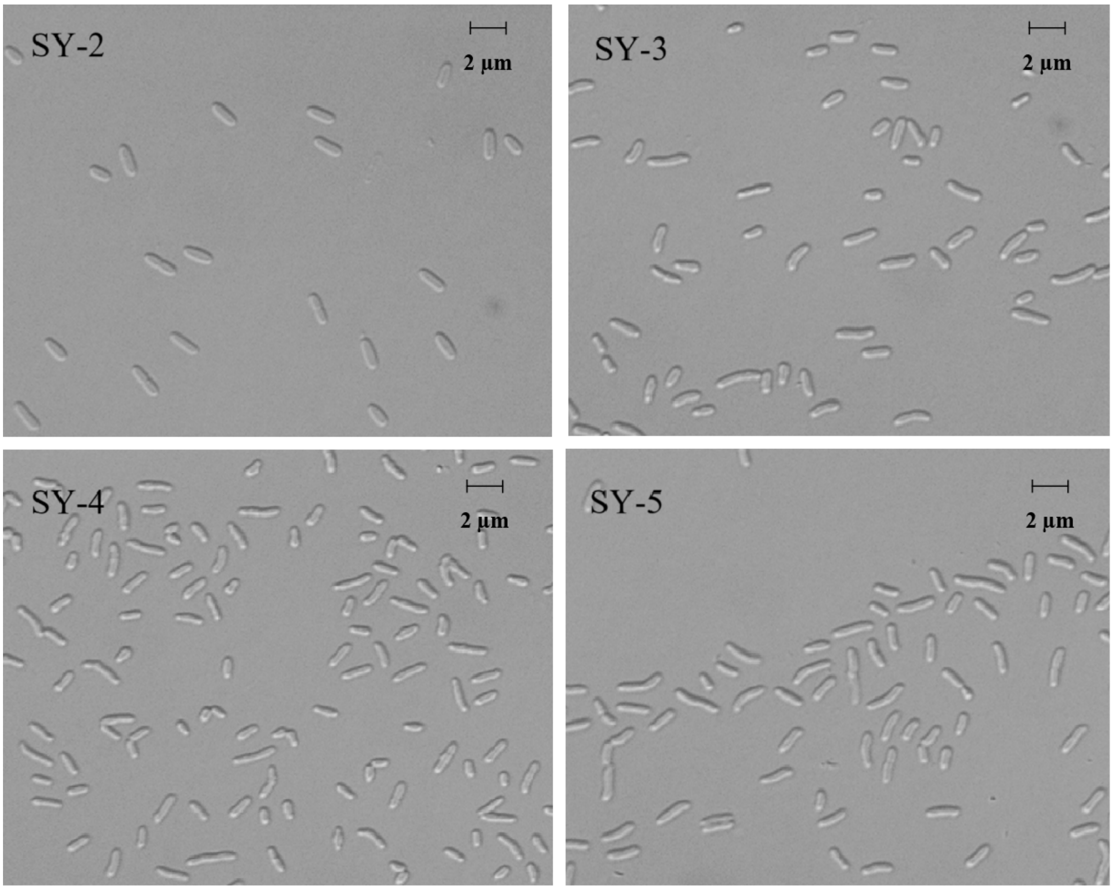

Figure. S1 Microscopic view of petroleum-degrading strains

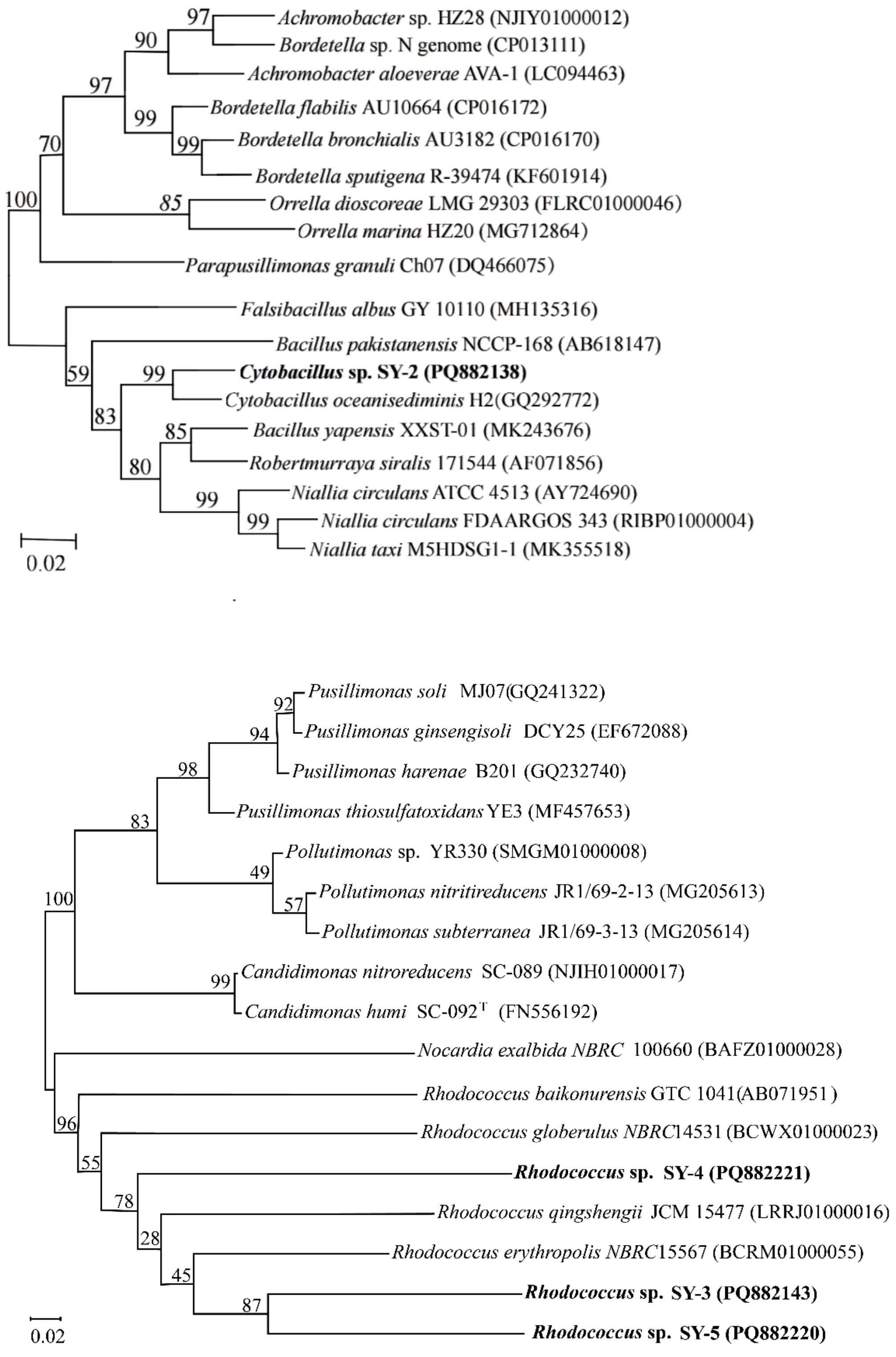

Figure. S2 Neighbor-joining tree of strains based on 16S rRNA gene

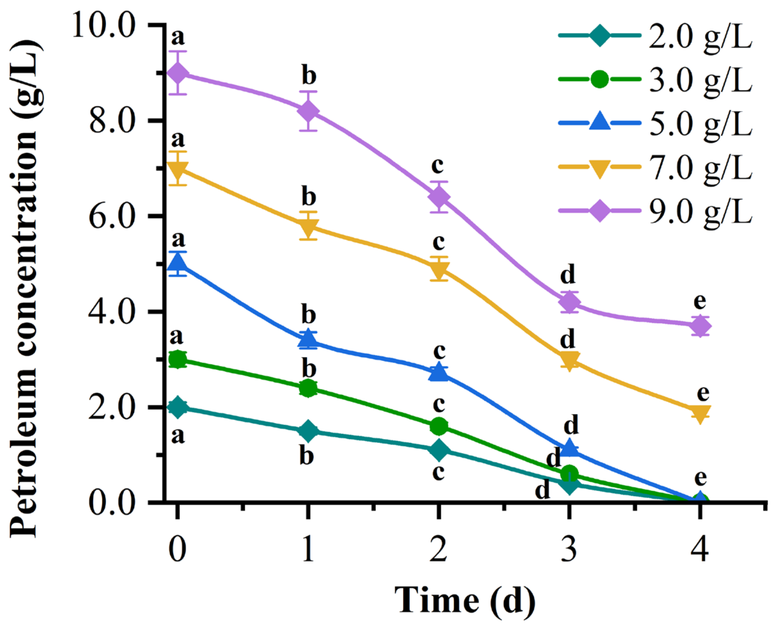

Figure. S3 Effect of initial petroleum concentration on degradation by microbial consortium. Groups with different lowercase letters at each concentration are significantly different (P<0.05, n=3).

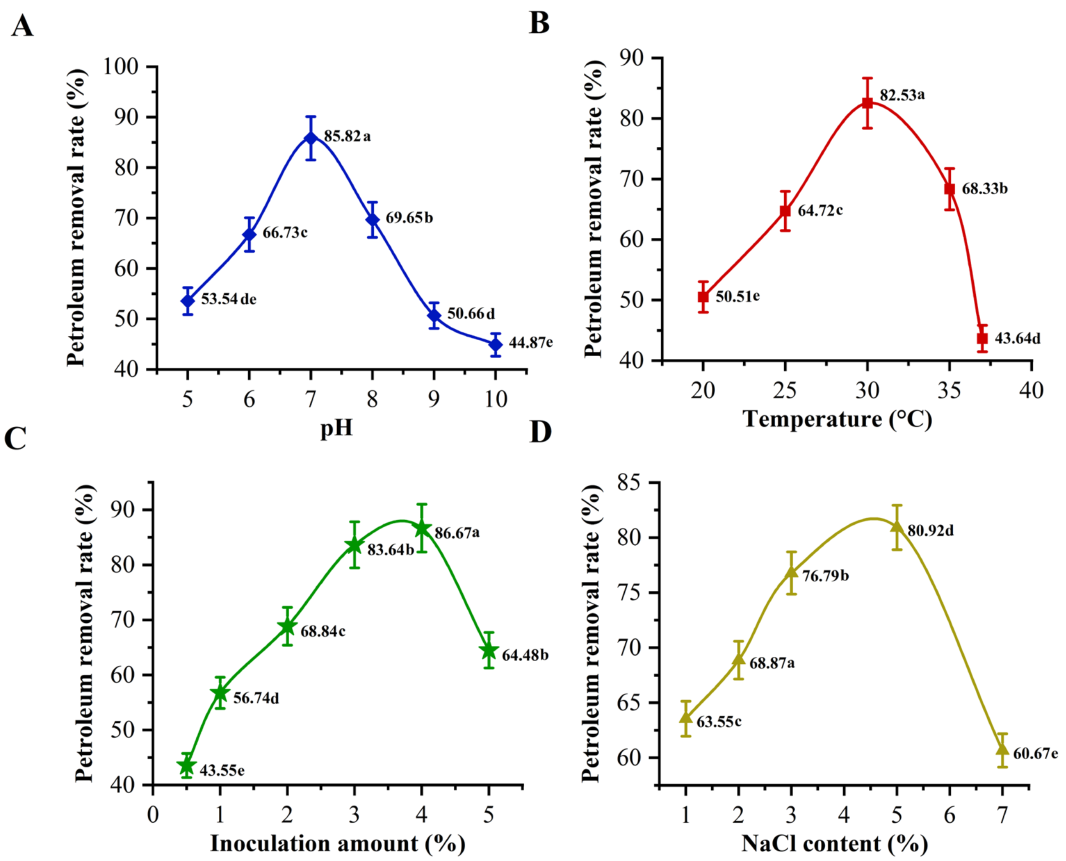

Figure. S4 Effects of different influencing factors on the degradation of petroleum-degrading microbial consortium.

A: pH; B: Temperature (°C); C: Inoculation amount (%); D: NaCl content (%). Groups with different lowercase letters are significantly different (*P*<0.05, n=3).

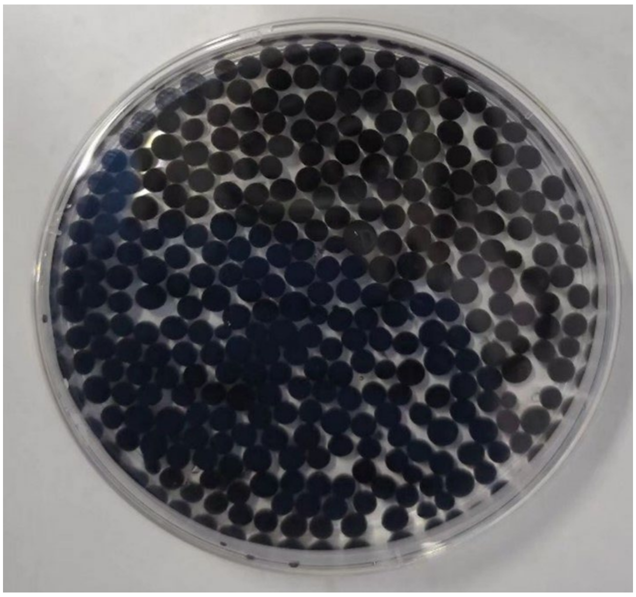

Figure. S5 Sodium alginate-biochar microspheres

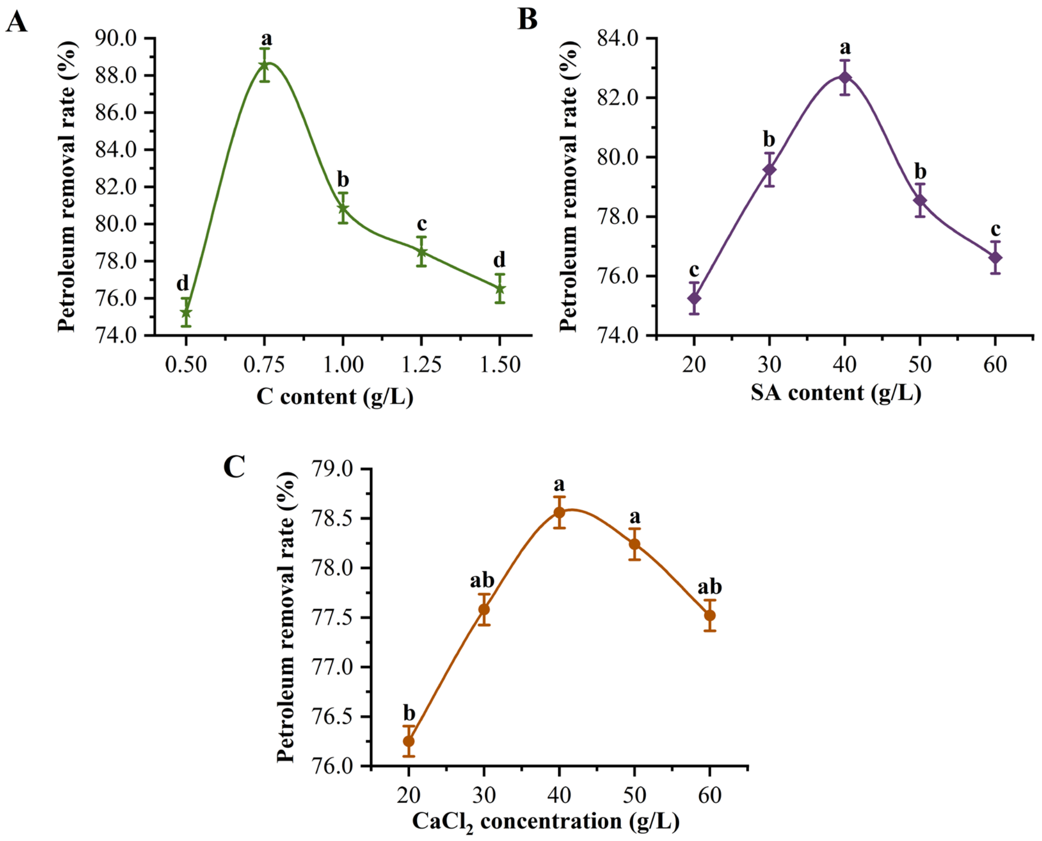

Figure. S6 The effects of biochar, sodium alginate, and $CaCl_2$ concentration on the removal of petroleum by immobilized microspheres.

A: Biochar content ($g·L^{-1}$); B: Sodium alginate (SA) content ($g·L^{-1}$); C: $CaCl_2$ concentration ($g·L^{-1}$). Groups with different lowercase letters are significantly different ($P<0.05$, n=3).

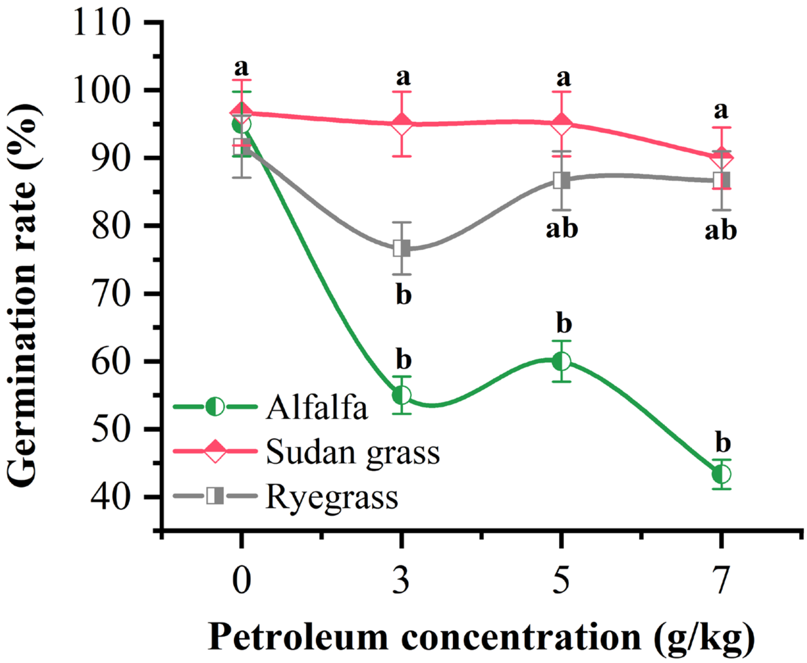

Figure. S7 Germination rate of three tested plants in petroleum-contaminated soil. Groups with different lowercase letters are significantly different (*P*<0.05, n=3).

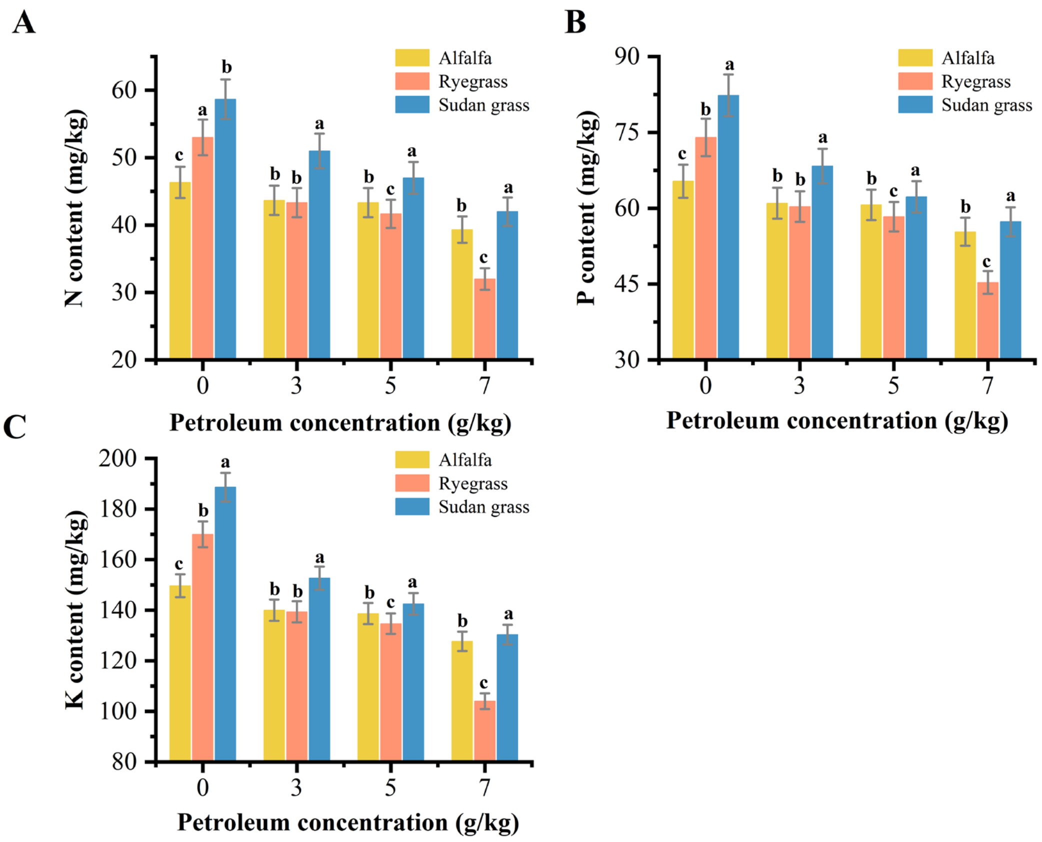

Figure. S8 Physicochemical properties of tested plants in petroleum-contaminated soil. A: N content (mg·kg^{-1}); B: P content (mg·kg^{-1}); C: K content (mg·kg^{-1}). For each plant species, groups with different lowercase letters at the same petroleum concentration are significantly different (*P*<0.05, n=3).

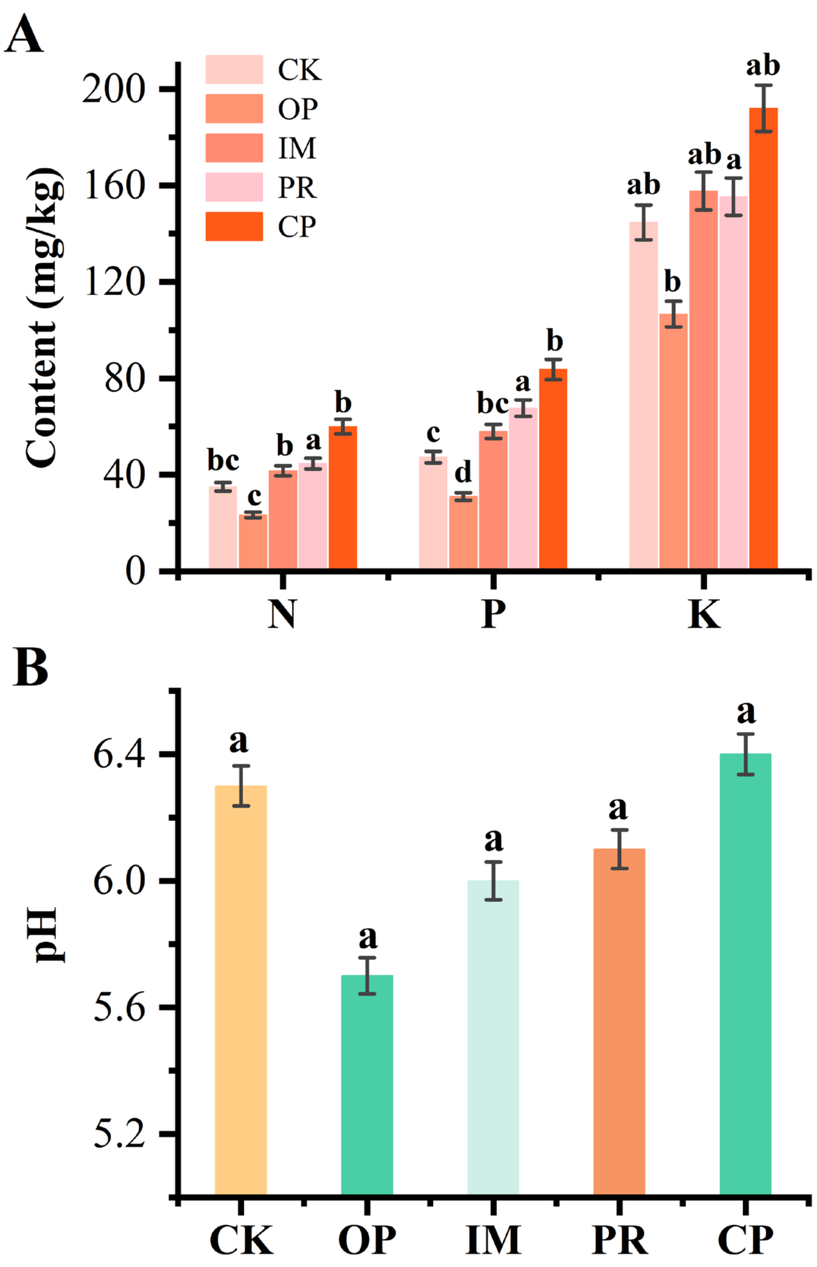

Figure. S9 Physicochemical properties of soil under different treatments

A: Contents of nitrogen (N), phosphorus (P), and potassium (K) in soil under each treatment;

B: Soil pH under each treatment.

For each nutrient (N, P, K, pH), groups with different lowercase letters are significantly different ($P<0.05$, n=3).

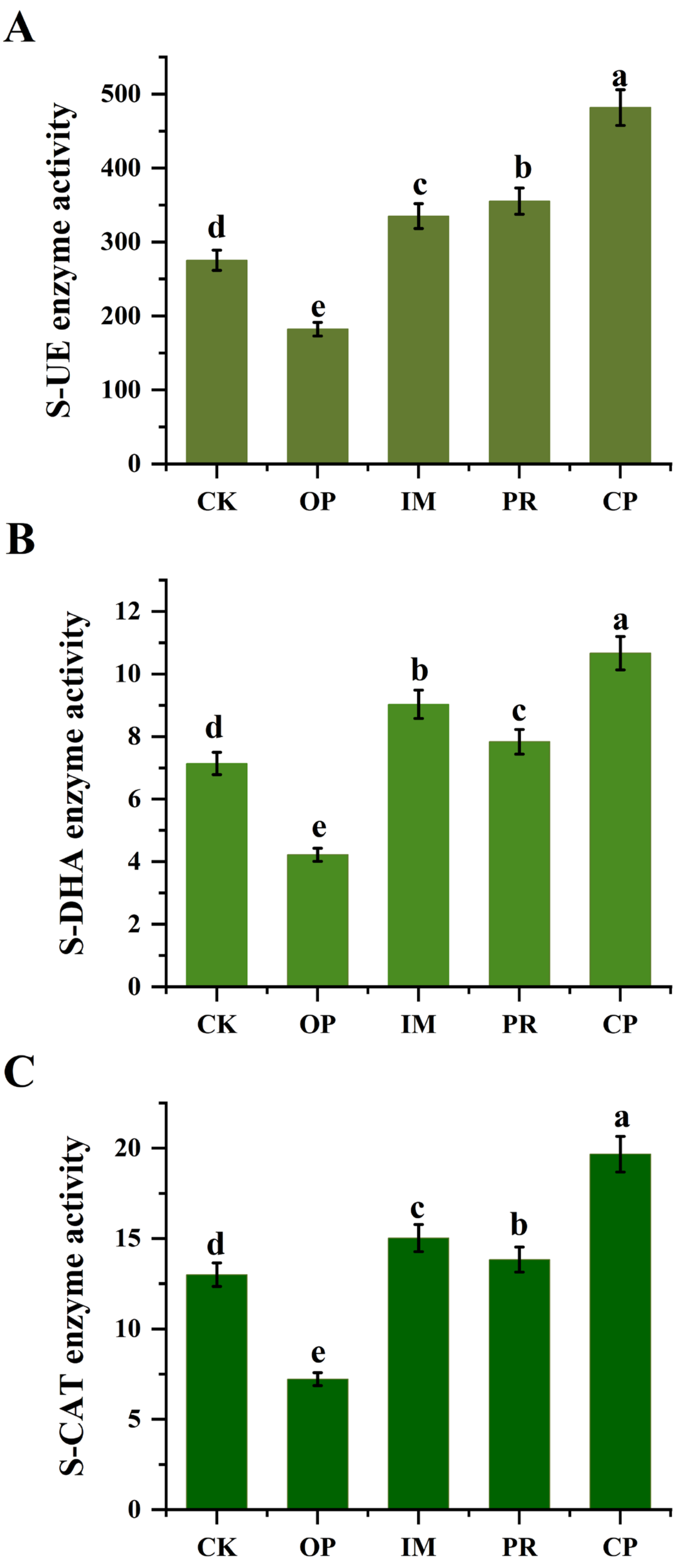

Figure. S10 Effects of different treatments on soil enzyme activity.

A: Urease (S-UE) activity; B: Dehydrogenase (S-DHA) activity; C: Catalase (S-CAT) activity.

Groups with different lowercase letters are significantly different (*P*<0.05, n=3).

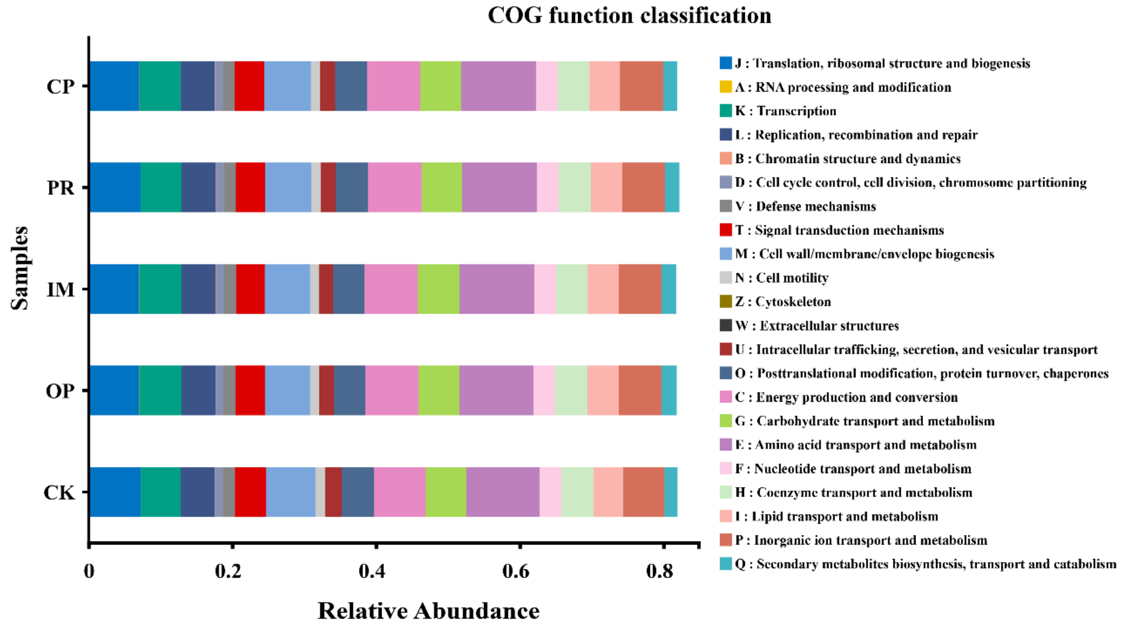

Figure. 11 Prediction of bacterial function in different soil samples
